# Diffusion Path Identification of Public Opinion Involving Enterprise Green Technology Adoption: An Interpretive-Structural-Modeling-Based Approach

**DOI:** 10.3390/ijerph19052817

**Published:** 2022-02-28

**Authors:** De Xia, Nian Xia, Yishi Zhang, Jiwei Xiong, Ruilin Zhu

**Affiliations:** 1 School of Management, Wuhan University of Technology, Wuhan 430074, China; xiade@whut.edu.cn (D.X.); xianian@whut.edu.cn (N.X.); 2 School of Management, Jinan University, Guangzhou 510632, China; xiongjiwei@stu2019.jnu.edu.cn; 3 Management School, Lancaster University, Lancaster LA1 4YX, UK; ruilin.zhu@lancaster.ac.uk

**Keywords:** green technology adoption, public opinion, interpretive structural model

## Abstract

With the increasing information transparency of business operations’ environmental influences, public opinion plays an important role in the green technology adoption of enterprises. Identifying the diffusion path of public opinion involving the process of enterprise green technology adoption is a significant task to verify the triggering mechanisms among the external factors and internal ones. An appropriate framework may help to clarify how the sustainability elements of public opinion are introduced to green technology adoption. Therefore, an interpretive structural-modeling (ISM)-based approach was applied to explore the basic transmission process and path of public opinion involving green technology adoption in enterprise practices. From the pressure of public opinion to the stakeholders involved, as well as the corresponding operational environmental activities, this study explored the psychological behavior of internal and external stakeholders and tried to clarify what the driving elements of green technology adoption are and how they relate to each other. Based on the field data collected from practitioners with Chinese contextual experience, the driving elements of the enablers of green technology adoption by enterprises were identified, and the fundamental triggering mechanisms of the public opinion pressure among them were analyzed. Thereafter, the influence of internal and external stakeholders involving green technology adoption and their corresponding behaviors under the pressure of public opinion were determined and expounded comprehensively, which illustrates the diffusion path of how public opinion influences the operational green technology adoption. This may narrow the gap between public environmental expectation and business operations. Finally, the managerial implications and the limitations of this study were concluded. The explanatory corresponding ISM model established in this study enriches the literature on the theoretical research of the mechanisms of green technology adoption.

## 1. Introduction

With the boom traditional industry and wealth, the ecological environment has been degraded dramatically, resulting in many negative impacts, such as resource exhaustion and serious pollution [1]. Over the past few decades, emerging public environmental consensus about environmental issues has significantly led enterprises’ attention to sustainable development [2]. Facing the deteriorated ecological environment, the public has expressed intensive and heavy concern about environmental destruction. Increasing public concern about environmental protection has promoted the corresponding information transparency. The environmental information about business operations may also be of great concern for consumers, governments, and enterprise managers and will affect daily operational innovation including technology adoption. Therefore, compulsory regulations and codes promote the adoption of green technology by enterprises, and this is gradually becoming a concerning factor for practitioners. Encountering strong public opinion and uncertain environmental problems, the governments, who play the role of “watchdog” regarding environmental protection, have issued a series of industrial policies, regulations, and norms. They encourage enterprises to adopt green technology with guidance and specifications for practitioners [3].

Regarding the influence of public opinion, green technology adoption (GTA) in the operational sector is a multi-dimensional decision-making process involving internal and external stakeholders. The challenge that policy designers and enterprises face is identifying the potential enablers, the influencing diffusion path, and the transferring network of GTA among internal and external operational stakeholders [4]. However, the extant research on GTA focuses mainly on the macro-industrial level; although some researchers have focused on the operation level, they have been limited to the application of green technology and resource consumption. How the external elements influence the operational activities for enterprises and whether the correlation among the enablers of GTA exists are still open problems, which makes overcoming the barriers in sustainability campaigns very difficult for the policy designers and the operational practitioners with green ambitions. Therefore, it is imperative to explore a clear map of the triggering and promoting mechanisms of external public opinion for the GTA of enterprises for sustainable operation.

To this end, this study attempted to explore the diffusion framework of public opinion involving the process of GTA in the enterprises, trying to answer how public opinion influences the intention and behavior of key internal and external stakeholders with the fundamental transmission process and diffusion path involved in the GTA of businesses. It is necessary to explore the driving elements and enablers of GTA in the identification of the stakeholders and their role, so as to clarify the interactive relationship among them. The linkage of GTA elements and the relationship network among them are the key factors to figure out the diffusion mechanism of public opinion in GTA. In this study, a comprehensive ISM was applied to that end [5]. According to [6], the reliability of ISM, an effective method to explore the construct of the mechanism, has been widely confirmed by peer research [7,8].

**Figure 1:**
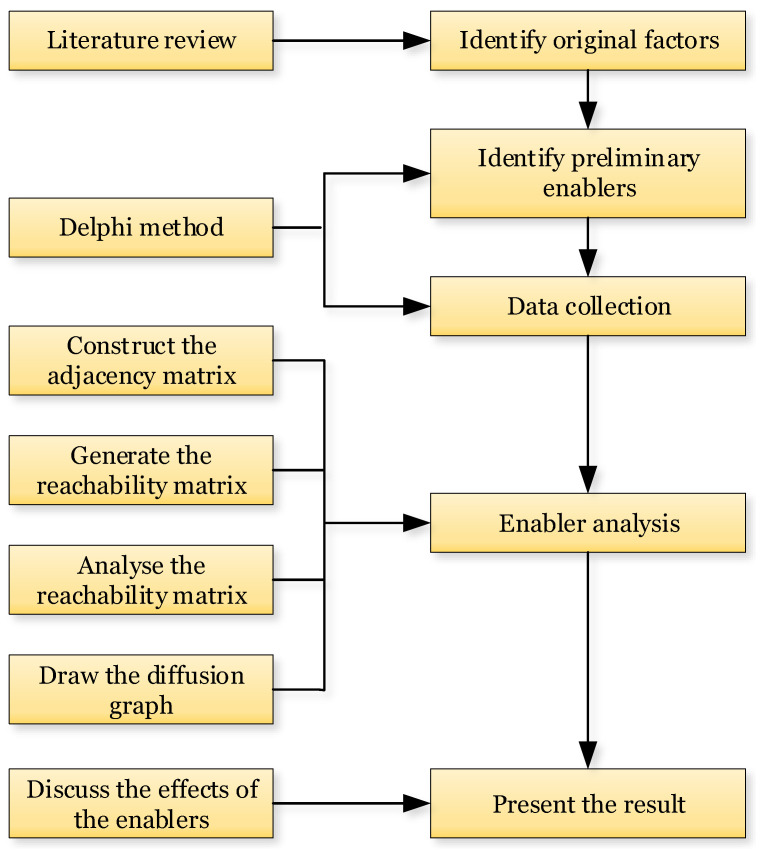
The overarching framework of this study.

This paper is organized as follows: After introducing the research background, the conceptual definition of the scientific issues is introduced for the goal of the work. Then, significant enablers of GTA are explored and analyzed in Section 2. In Section 3, the Delphi method is applied with existing empirical findings and our theoretical explorations to verify the preliminary enablers. The ISM is then established and explored to evaluate the relative importance of the enablers and their interrelations. Section 4 analyses the results and clarifies the triggering logic and path of public opinion pressure diffusion among the enablers of GTA. In Section 5, the theoretical contributions, the managerial implications, and the limitation of this study are finally concluded. The overarching framework of this study is presented in Figure 1.

## 2. Preliminary Screening of Enablers of GTA

Green technology refers to processes and technologies that reduce material and energy consumption [9]. Herein, green technology adoption (GTA) focuses on the introduction of green technology to business operations by enterprises, especially during new product development, production processes, and the training system for the human resources. This study mainly focused on the timing, degree, and influencing factors of GTA. Del Río González took the Spanish paper industry as an example and argued that the adoption of green technology is influenced by external factors including environmental policies, suppliers, consumers, competitors, NGOs, financial departments, and R&D centers, internal factors including enterprise characteristics, organizations, technology, and environmental strategies, and the characteristics of the technology itself [10]. Thiruchelvam et al. pointed out that the adoption of green technology by small- and medium-sized enterprises would be restricted due to the lack of education, training, organization, and coordination ability [11]. Garrone et al. argued that the adoption of advanced wastewater treatment technologies is influenced by the factors at the company and the community levels, where the former include knowledge assets, environmental technology, and industry partners and the latter include citizens’ voice and the government’s environmental regulations [12]. Bharati and Chaudhury believe that the top management and consumers of an organization will have an important impact on the adoption of green technology in enterprises [13]. Yao et al. took medium and micro-enterprises as their research object and concluded that, driven by market forces, the green supply chain, green practice, green consumption, and green innovation become the endogenous driving forces for SEMs to adopt green technology [14].

With the previous exploration of green behavior and technological innovation, there are several enablers implied during the process of GTA.

### 2.1. Public Opinion with Environment Information Disclosure

Currently, the increasing transparency of environmental information makes public appeals more focused, prodigious, and penetrating. Regarding the green practices of enterprises, the extant literature reveals the importance of public opinion in the diffusion of sustainable technologies and the resultant commercial applications [15]. From the perspective of public participation in virtual communities, Sichani and Jalili [16] pointed out that individuals who are extremely dispersed in society will quickly group because of their common interests or similar opinion. They will express identical ideas or take similar actions to force the government, enterprises, and social organizations to listen to their “voices”. Hence, public opinion essentially reflects the interests, aspirations, and requirements of certain communities and groups. Logically, group consciousness is the fundamental basis of enterprise legitimacy, which influences business operations. Public consensus on environmental events or issues reflects the public’s moral preferences, environmental awareness, and value orientation. One of the critical reasons for enterprises to pay attention to environmental protection is the social pressure resulting from public opinion intensively displayed by media or environmental protection groups. Meanwhile, the influence of operational groups may accelerate the application of environmental technologies in business operations. Internal and external pressures are also caused by the environmental awareness and moral values of the public and the average person, as a force that can regulate individual and organization behavior. This encourages the government to strengthen the information disclosure of enterprise operations, promote enterprises to fulfill their social responsibilities, and motivate public participation [17]. Therefore, public opinion was regarded in this study as a key element affecting the players’ behaviors and the GTA of enterprises.

### 2.2. Environmental Policy

Environmental protection for the welfare of society, directly or indirectly, is the vital content of environmental policy. It affects the behavior of enterprises, as well as relevant individuals. The regulations and codes issued by the government are the long-term approaches to regulate and guide the enterprises’ behaviors in the value-added activities of businesses, especially when the market mechanism does not work properly in certain fields [18]. Environment policies built with the consensus and mission of public opinion (which contains collective willingness and vision) are important signals and tools to intervene in operational decision-making, coordinate action, and meet the expectations of the future. The benefit of standing by environmental policy, including taxes, customs, and extensive costs, will steadily encourage enterprises to adopt green technology in the long term [19]. It may also reduce the possibility of government intrusion and consolidate the legitimacy of business operations by fulfilling the environmental responsibilities [20]. Morally, GTA may help enterprises mitigate the pressure from the government, as well as environmental organizations, maintain a positive effect, and obtain support from outsiders. So far, multi-agent and multi-policy tools may be applied to boost GTA [21]. They respond to the ecological needs of the whole society and encourage production and service entities to adopt green technology. For consumers, environmental policy can be regarded as a heuristic rule by which consumers will maintain more sustainability behaviors in their daily lives, such as waste classification, ecological label preference, and a low-carbon lifestyle. Therefore, under the situation of the intensive influence of public opinion and disclosure of environmental information, relevant policies issued by the government are regarded as a powerful factor in enterprise operations. The positive interaction between environmental policies and enterprises’ GTA is conducive to achieving a “win-win” status for environmental protection and economic development.

### 2.3. Sustainability Beliefs of Top Managers

Top managers’ belief in environmental protection is fostered by a combination of environmental factors such as policies, partners, and customers. The task-oriented circumstance would affect the understanding and assessment of environmental problems, which would be incorporated into the production and other operational activities of the enterprise. As the decision-makers of an organization, the psychological state and value orientation of the top managers are the fundamental basis for developing a sustainability strategy for the organization [22]. The beliefs and commitments of top managers are the primary driving force behind organizational decisions and strategies. In the context of public opinion and environmental issues, top managers and their teams may recognize that the alignment of organizational behavior with the public’s morals and values is the fundamental basis for organizational survival and growth [23]. It can therefore be argued that the beliefs and ambitions of top managers play a critical role in organizational behavior towards sustainable development. Besides, existing research also reveals that the psychology and behavior of decision-makers and technicians are significantly related to the features and style of the technology adopted [24]. Therefore, top managers’ beliefs should be taken into account as an important element of GTA.

### 2.4. Consumers’ Green Awareness

The sustainability of consumption has become an important topic and even a popular value for the public, which constantly influences and shapes consumers’ perceptions and their daily lives [25]. In general, consumer preferences for sustainability in consumption may involve some additional costs (e.g., extra time, emotion, patience, and behavioral change), although the behavioral change may benefit society and the public’s welfare. Therefore, except for the psychological support for environmental protection, consumers always buy conventional products rather than green products. There are many factors that may cause the gap between the public expectation and the individual practical choice, including habits, compatibility, and high prices. However, with the seriousness of environmental pollution, the government and related organizations have paid close attention to environmental issues, trying to promote the consumption of green products through environmental policies, price subsidies, and media campaigns. As a result, a positive circumstance for GTA may develop in the end market. This helps consumers think twice and gradually recognize and accept the value of green product consumption for environmental friendliness and the public’s welfare [26]. The social symbolic function of green products as a label also psychologically satisfies the environmental ethical needs of consumers. By purchasing green products, consumers want to express their concern for the environment and ecology, and at the same time convey a positive and responsible attitude. In this way, consumers raise social awareness as a whole. Therefore, consumers’ sustainability preferences determine the characteristics and prices of the products [27], guiding enterprises to design and produce green products for consumers. Based on the above analysis, we propose that consumers’ green awareness plays an important role in shaping enterprises’ sustainability behavior.

### 2.5. Financial Subsidies

Subsidies and related support in the form of direct or indirect transfer payments of public funding for green production and green products are the fundamental way to overcome the high prices that arise from the application of green technologies in practice. The willingness of the public to promote environmental protection, especially as an expression of public opinion, is reflected by an exclusive budget for a government subsidy program for green ambitions. Enterprises standing up for the green campaigns may gain significant support for sustainable development. Insufficient subsidies for different fields (such as consumers, enterprises, green products, etc.) are considered as a barrier to the diffusion of green technologies [28]. Subsidies are necessary for participants to overcome the economic and non-economic barriers. Firstly, they benefit governments, enterprises, and consumers, who may have the willingness to understand the environmental issues, but have very limited budgets. With the very special planning of budgets regarding deference to green innovation, they help overcome the price disadvantages and increase consumer enthusiasm [29]. Cohen et al. showed that subsidies for green products with environmental attributes can promote the adoption of green technology in enterprises and their consumption in the end markets, balancing the concerns from the environmental and economic aspects [30]. Appropriate subsidies generate innovation and help enterprises achieve sustainable operations [31]. Nie and Yang also argued that subsidies provide an endogenous self-participation incentive for enterprises [29]. This is more flexible and task-oriented as an initiation tool to promote green behaviors, compared with the environmental policies and laws with a long-term horizon, even though they may be oriented toward the same end. Therefore, we regarded financial subsidies in this study as a potential key enabler of GTA.

### 2.6. Consumers’ Willingness to Pay

In addition to the traditional physical function, consumers’ willingness to pay for green solutions is boosted by the value and utility in many ways. One is the benefit from the subsidies; the other is the moral and ethical demand cultivated in certain communities with public consensus. Regarding green products, the utility is presented as a unique environmental attribute. The value of the environmental attribute may be self-interest or altruism. Consumers decide whether to pay or not after self-evaluation based on their own beliefs. The strong environmental public opinion reflects the extensive demand for a healthy environment as a public good. Positive public opinion, in turn, prompts governments and society to foster green consumer behavior through a series of means such as subsidies and media support. Consumers’ awareness of environmental friendliness can greatly stimulate their interest in and demand for green products [32]. Some studies have shown that consumers are willing to pay extra for the sustainability characteristics of products (e.g., [27]), which shapes the beliefs of top managers about sustainable operations and their market reputation.

### 2.7. Sustainability Indicators of Business Performance

Sustainability indicators built into business operations are the signals of GTA. They are the concrete responses in greening the operations of enterprises, customers, and governments with sustainability value and vision. Facing the growing social concerns of the public, the enterprises’ response is to develop sustainability indicators within the management of operational performance to boost the implementation of a sustainable triple-bottom line, namely the interaction of three dimensions of sustainability. In recent years, the implementation of enterprises’ management of sustainable performance has been introduced as the main characteristic. However, enterprises are still subject to public accusations and complaints as they fall short of public expectations. Therefore, enterprises make efforts to embed sustainability in the management of operational performance through systematic sustainability indicators. Some scholars have even suggested the integration of environmental and social measures into the management of operational performance [33]. Nudurupati et al. argued that a series of measures with indicators is necessary to monitor and manage performance across multiple dimensions, which facilitates the practice of green operations [34]. It forms intuitive green behaviors at different levels with detailed goals and guidelines for specific aspects such as cost management, human resources, and quality management.

Meanwhile, studies have shown a positive correlation among financial results, social welfare, and environmental performance [35]. The positive impact of environmental performance on green production and green products helps enterprises gain competitive advantage and enhance sustainability. Social performance is one of the three dimensions that cannot be ignored [36]. As a pillar of sustainability, the performance indicators of the social dimension can reflect the status of corporate social responsibility and the enthusiasm to adopt green technologies.

The key enablers of GTA summarized above are listed in Table 1.

## 3. Identification of the Relations among Enablers

### 3.1. Verification of the Enablers Using the Delphi Method

In this section, the Delphi method is applied to validate these enablers and to obtain the relationships among them. Anonymous survey letters were sent to respondents to explore their perspectives. Professionals with more than three years of corresponding work experience were invited to participate in interviews to validate the driving elements and the relationships among them. A total of 150 senior professionals of 56 Chinese firms from the intensive energy and resource consumption industries, such as steel, civil engineering, and auto industries, were selected for the survey, of which 126 surveys were validly collected. Detailed information on these professionals is given in Table 2.

With extensive literature reviews and the empirical study above, the correlations among various driving elements and GTA were explored and verified, and a preliminary conclusion could thus be obtained. The investigation results are shown in Table 3. It was observed that 83.33% of the professionals believed that public opinion with environment information disclosure is related to GTA. Besides, all of the professionals confirmed the seven elements as enablers, although there was some variation regarding the significance of the influence.

### 3.2. Constructing the Diffusion Network of the Enablers Involving GTA Using ISM

The contextual relationships between each pair of elements were identified through the “VAXO” matrix formulation with experts’ opinions. In this study, 126 valid experts in fields related to GTA in enterprises were interviewed. In the beginning, the goal of the study was to introduce them to an explanation of the meaning of the matrix and the rules to be followed when the interview was carried out: “V” represents that E${}_{i}$ leads to E${}_{j}$, but E${}_{j}$ does not lead to E${}_{i}$; “A” represents that E${}_{i}$ does not lead to E${}_{j}$, but E${}_{j}$ leads to E${}_{i}$; “X” denotes that E${}_{i}$ and E${}_{j}$ lead to each other; “O” represents that E${}_{i}$ and E${}_{j}$ are not related to each other. For each pair of elements, the first check predicts the relationship based on “Yes (Y)” or “No (N)”. If the answer was “Yes”, further explanation was requested and helped us obtain the “Interpretive Logic Knowledge Base”, as shown in Table 4.

Hereafter, the interpretative structural model (ISM) was applied to explore more details of the relations among enablers. ISM is a widely-used tool for complex system analysis [45]. It is generally felt that individuals or groups encounter difficulties in dealing with complex problems or systems. The difficulty or complexity of a system is due to the large number of elements and the interactions among them. ISM is a well-established methodology to identify the relationships among specific items. For example, Raj et al. explored the interaction among the driving factors of a flexible manufacturing system (FMS) through ISM and distinguished “driving enablers” (i.e., influencing the other enablers) and “dependent enablers” (i.e., influenced by enablers) [46]. Mathiyazhagan et al. used ISM to identify the barriers for enterprises to implement green supply chain management and explored the mutual influence and importance of 26 barriers, so as to help enterprises eliminate dominant barriers [47]. Poduval and Pramod used ISM to analyze the influencing factors of total productive maintenance (TPM) [45]. ISM contains an interactive process in which a set of directly and indirectly related elements are organized into an all-inclusive systematic framework. The steps of ISM development are described as follows:

Step 1. Identifying the system factors’ set. The factors of GTA were generated through the literature review and Delphi method, as shown in Table 1;

Step 2. Constructing the adjacency matrix *R*. The element $r_{ij}\in R,\left(i,j=1,2,...,7\right)$ can be originated and transformed from the corresponding element in Table 4. The transformation rules are listed below:

1. If i=j, $r_{ij}=r_{ji}=1$;
2. If $r_{ij}$ refers to V in Table 4, $r_{ij}=1$ and $r_{ji}=0$;
3. If $r_{ij}$ refers to A in Table 4, $r_{ij}=0$ and $r_{ji}=1$;
4. If $r_{ij}$ refers to X in Table 4, $r_{ij}=r_{ji}=1$;
5. If $r_{ij}$ refers to O in Table 4, $r_{ij}=r_{ji}=0$.

Then, the adjacency matrix of enablers for GTA could be established with the empirical information as shown in Table 5;

Step 3. Generating the reachability matrix. The reachability matrix was derived from the adjacency matrix with the transitivity considered. Transitivity links ensured the consistency of the links measured and ensured that all possible interpretations were included in the model: If $E_{i}$ leads to $E_{j}$ and $E_{j}$ leads to $E_{k}$, the path that $E_{i}$ leads to $E_{k}$ will be reasonable according to the transitivity principle. The process of the reachability matrix construction is shown in Table 6;

Step 4: Analyzing the reachability matrix. After the reachability matrix was obtained, the operational level among elements was explored regarding the diffusion path. Level partitioning is the process of ranking each element at different levels (see Table 7). The reachability set and antecedent set were obtained from the final reachability matrix. The reachability set $R\left(S_{i}\right)=\{$corresponding elements of all $r_{ij}=1$ in the $S_{i}$ row of the reachability matrix, j=1,2,...,N}, the antecedent set $A\left(S_{i}\right)=\{$corresponding elements of all $r_{ij}=1$ in the $S_{i}$ column of the reachability matrix, j=1,2,...N}.

**Figure 2:**
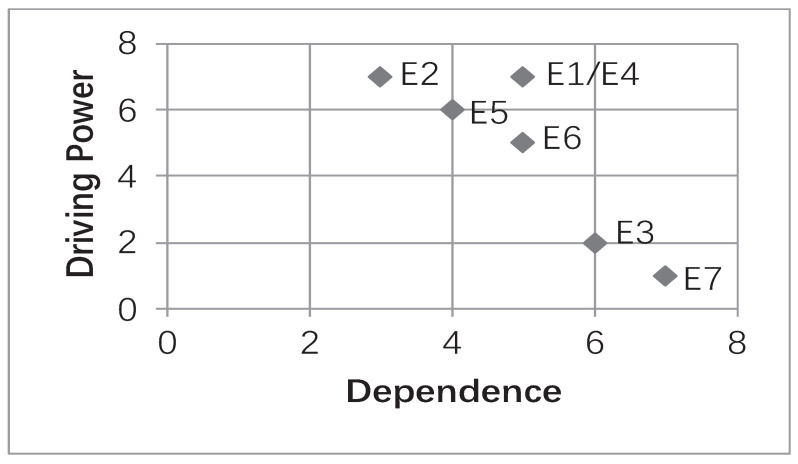
Level profile of enablers of GTA.

If the intersection of the reachability set and the antecedent set is the reachability set itself, these variables occupy the top level of the hierarchy, the intersection being represented by *C*, that is $C=R\left(S_{i}\right)\cap A\left(S_{i}\right)$. The rule defined at different levels was “if $S_{i}$ is the most advanced node, the following condition $C=R\left(S_{i}\right)\cap A\left(S_{i}\right)=R\left(S_{i}\right)$”. To obtain the subsequent level variables, the elements that were output were to be removed and executed repeatedly. According to the above rules, we successively concluded the nodes from the first to fifth levels of the enablers listed in Table 8. The dependency and driving power would lead to Figure 2 for the final hierarchy;

Step 5. Drawing the ISM relationships diagram. Following the results of partitioning the reachability matrix, the top-level factor was positioned at the top of the hierarchy, and the second-level factor was placed just below the top level. This process was repeated until the bottom-level factors were placed at the lowest position in the hierarchy.

## 4. Result Analysis and Discussion

The key elements and the influencing diffusion path are the critical contexts of the mechanism impacting the adoption of green technology in operations. Firstly, the significant operational elements of GTA were explored with theoretical analysis and an empirical study. Even though there is a growing number of studies on GTA, the diffusion path and network of the enablers of GTA need to be further studied. In this study, customers, the government, and top managers were the essential stakeholders with their characteristics and power. Herein, their significant roles and behaviors involving enterprise decision-making were analyzed from both the internal and external perspectives and were integrated into the comprehensive framework for GTA. Seven important elements of GTA and their relationships were verified and constructed, which were tied to top managers, customers, the government, and the public, directly or indirectly. In particular, the results also showed that there were differences between environmental policy and financial subsidies as the drivers regarding their transitive attributes and roles in the diffusion process, even though they (both belonging to public power) attempt to reach a similar green goal. The results of this study provide more details compared with the extant literature about the role of environmental policy during a green campaign [48]. In contrast to the indirect impact of environmental policy, financial subsidies influence consumers and the public directly, which boosts their green behaviors as a start-up tool with flexible responsiveness. Meanwhile, environmental policy exerts a direct influence on financial subsidies. This implies that, during the diffusion process, environmental policy and financial subsidies play different roles with the same purpose in the end, with their respective adjacency and transitive attributes under the particular circumstance.

**Figure 3:**
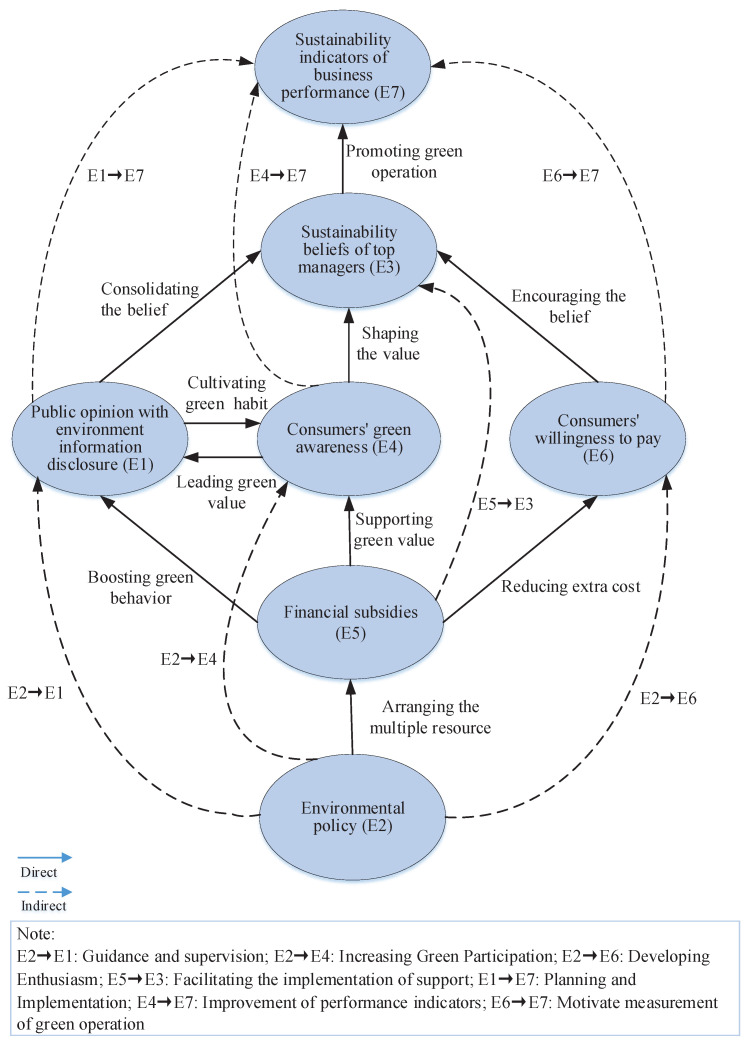
The diffusion network of the enablers of GTA.

The influencing diffusion path was also explored and is shown in Figure 3. It can be observed that public opinion with environment information disclosure, financial subsidies, and environmental policy are the significant driving forces producing internal and external pressure on enterprises in a green campaign. Furthermore, the framework with the hierarchy illustrates the logic among the elements in the dynamic system. Here, public opinion with environment information disclosure (E1), consumers’ green awareness (E4), and consumers’ willingness to pay (E6) are at the same level within the framework. This indicates that regarding the impact on GTA, these three elements associated with the critical stakeholders may have the same transitive location during the diffusion process by influencing the sustainable beliefs of top managers (E3). Meanwhile, E3 is the only node that transfers and diffuses the previous impact to the end of the system. It can be noted that the framework begins with the environmental policy (E2) instead of public opinion at the first level (shown in Table 8), which reminds us that the environmental policy may be the antecedent of public opinion diffusion leading to green operations. As a whole, the hierarchical framework displays the fundamental diffusion path and transitive logic that links essential factors from various stakeholders in green operations under public expectation.

Eventually, the relationship among these hierarchical factors are illustrated with direct and indirect linkages. The direct linkages (impact) including bilateral and unilateral relationships play a fundamental role among the seven drivers in a certain order. It can be observed from Figure 3 that the bilateral relationship is represented by the linkages between public opinion with environmental information disclosure (E1) and consumers’ green awareness (E4), indicating that they promote and boost each other during the diffusion process. Although most of the linkages are unilateral, they constitute an explicit diffusion order with clear dependencies among the drivers. Associated with direct linkages, the indirect ones are also included and displayed in the network, implying their primary and secondary role in the transitive sequence.

The results also showed that consumer’s green awareness (E4), consumers’ willingness to pay (E6), and public opinion with environmental information disclosure (E1) are very sensitive elements with a strong dependency, while financial subsidies are the fundamental driving force. It influences the three succeeding factors (E1, E4, E6) directly and significantly and the descendant factor (i.e., sustainable beliefs of top managers (E3)) indirectly. The results in Table 8 also show that it is the top manager who can build the public voice and expectation into operational GTA. Therefore, top managers play a direct and decisive role in the adoption of green technologies and the indicator establishment of the sustainable performance of the business. Functionally, the performance profile of the sustainability indicators of the organization is the very signal of the business orientation guiding the investment and daily operation, which is also influenced indirectly by public opinion (E1), consumers’ green awareness (E4), and consumers’ willingness to pay (E6), simultaneously.

## 5. Conclusions

In this study, the elements contributing to GTA as enablers were determined based on stakeholder theory, enriching the literature in the field of enterprise’s sustainable development within the Chinese context. The theoretical framework showed the diffusion path with the map of the interrelationships among elements involving GTA, and the fundamental transitive mechanism was illustrated with hierarchical levels, which display the different roles regarding their adjacency and dependence attributes during the diffusion process. After seven relevant elements as GTA enablers were verified, the interrelationships during the influence diffusion path among the elements were explored with a hierarchical network. Herein, three types of relationships among them were identified, namely the direct unilateral, the direct bilateral, and the indirect influences. This study provided details about the drivers’ various functions during the process of public opinion diffusion and green technology adoption, theoretically contributing to the influencing diffusion mechanism and application of green technology in operational management.

The influencing diffusion path involving the enablers may help to understand the interrelationships among the elements within the framework during GTA. It may help top managers shape green strategies in business. For designers of public policies, it may facilitate reasonable and effective green codes and regulations to promote sustainable development.

Despite the possible contributions, the limitations of this study lied in the following aspects: the results of this study are sensitive to the samples, and their are shortcomings to the methodology of using questionnaires and interviews regarding the differences in the economic and education development of society, even though it has been applied in many previous studies. More reasonable methods such as scenarios and experimental methods carried out under different circumstances may help to mitigate these defects and may even provide access to the relative weight of the elements of GTA.

## Figures and Tables

**Table 1:** The driving elements in GTA.

Index	Element	Definition	Source
E1	Public opinion with environment information disclosure	Consciousness, interests, aspirations, and concern of certain community and social groups.	Cho et al. (2012) [37]
E2	Environmental policy	LicenseTaxCustoms duties	Kitzmueller & Shimshack (2012) [38], Song et al. (2019) [39]
E3	Sustainable beliefs of top managers	The psychological state, value preference, and ambition of top and senior managers	Dubey et al. (2015) [7], Meramveliotakis & Manioudis (2021) [40]
E4	Consumers’ green awareness	Consciousness about and concern for the environment and ecology in sharing values and green habits	Shaw et al. (2016) [41], Moser (2015) [42]
E5	Financial subsidies	Governments internalize the environmental costs of consumers and producers	Cohen et al. (2015) [30], Wang et al. (2017) [31]
E6	Consumers’ willingness to pay	Willingness to pay extra for green products	De-Magistris & Gracia (2016) [43]
E7	Sustainability indicators of business performance	Sustainability indicators of investment, production, marketing, and other operational contexts	Ahi & Searcy (2013) [44], Shibin et al. (2018) [6]

**Table 2:** Individual respondents’ statistics from the questionnaires.

Feature	Range	Frequency	Percentage (%)
Education	High school senior	26	20.6
	Undergraduate degree	64	50.8
	Graduate degree	36	28.6
Age	25–30	32	25.4
	31–40	26	20.6
	41–50	35	27.8
	51–60	24	19.1
	>60	9	7.1
No. of years employed	3–4	29	23.0
	4–6	31	24.6
	6–9	27	21.4
	9–12	34	27.0
	>12	5	4.0
Position	Director	21	16.7
	General manager	26	20.6
	Senior manager	42	33.3
	Senior staff	37	29.4
Type of firm	Steel	2	3.6
	Auto industry	23	41.1
	Electronics	8	14.2
	Civil engineering	12	21.4
	Energy and chemical	9	16.1
	Other	2	3.6

Notes: There were 126 valid respondents among 150 requested from 56 sectors.

**Table 3:** Summary of elements to be identified as enablers.

Index	Element	Relevant	Irrelevant
E1	Public opinion with environment information disclosure	83.33%	16.67%
E2	Environmental policy	100%	0%
E3	Sustainability beliefs of top managers	95.24%	4.76%
E4	Consumers’ green awareness	73.81%	26.19%
E5	Financial subsidies	88.10%	11.90%
E6	Consumers’ willingness to pay	76.19%	23.81%
E7	Sustainability indicators of business performance	92.86%	7.14%

**Table 4:** Structural self-interaction matrix.

	E7	E6	E5	E4	E3	E2	E1
E1	V	V	V	X	V	X	X
E2	V	O	V	V	V	X	-
E3	V	A	A	A	X	-	-
E4	V	X	A	X	-	-	-
E5	V	V	X	-	-	-	-
E6	O	X	-	-	-	-	-
E7	X						

**Table 5:** The adjacency matrix of GTA.

	E7	E6	E5	E4	E3	E2	E1	Driving Power
E1	1	1	1	1	1	1	1	7
E2	1	0	1	1	1	1	1	6
E3	1	0	0	0	1	0	0	2
E4	1	1	0	1	1	0	1	5
E5	1	1	1	1	1	0	0	5
E6	0	1	0	1	1	0	0	3
E7	1	0	0	0	0	0	0	1
Dependence	6	4	3	5	6	2	3	

**Table 6:** The reachability matrix of GTA.

	E7	E6	E5	E4	E3	E2	E1	Driving Power
E1	1	1	1	1	1	1	1	7
E2	1	$1^{\;a}$	1	1	1	1	1	7
E3	1	0	0	0	1	0	0	2
E4	1	1	$1^{\;a}$	1	1	$1^{\;a}$	1	7
E5	1	1	1	1	1	0	$1^{\;a}$	6
E6	$1^{\;a}$	1	0	1	1	0	$1^{\;a}$	5
E7	1	0	0	0	0	0	0	0
Dependence	7	5	4	5	6	3	5	

^a^ The transitive attribute.

**Table 7:** Level identification.

Iteration	Enabler	$R(S_{i})$	$A(S_{i})$	$R\left(S_{i}\right)\cap A\left(S_{i}\right)$	Level
1	E1	1,2,3,4,5,6,7	1,2,4,5,6	1,2,4,5,6	
	E2	1,2,3,4,5,6,7	1,2,4	1,2,4	
	E3	3,7	1,2,3,4,5,6	3	
	E4	1,2,3,4,5,6,7	1,2,4,5,6	1,2,4,5,6	
	E5	1,3,4,5,6,7	1,2,4,5	1,4,5	
	E6	1,3,4,6,7	1,2,4,5,6	1,4,6	
	E7	7	1,2,3,4,5,6,7	7	I
2	E1	1,2,3,4,5,6	1,2,4,5,6	1,2,4,5,6	
	E2	1,2,3,4,5,6	1,2,4	1,2,4	
	E3	3	1,2,3,4,5,6	3	II
	E4	1,2,3,4,5,6	1,2,4,5,6	1,2,4,5,6	
	E5	1,3,4,5,6	1,2,4,5	1,4,5	
	E6	1,3,4,6	1,2,4,5,6	1,4,6	
3	E1	1,2,4,5,6	1,2,4,5,6	1,2,4,5,6	III
	E2	1,2,4,5,6	1,2,4	1,2,4	
	E4	1,2,4,5,6	1,2,4,5,6	1,2,4,5,6	III
	E5	1,4,5,6	1,2,4,5	1,4,5	
	E6	1,4,6	1,2,4,5,6	1,4,6	III
4	E2	2,5	2	2	V
	E5	5	2,5	5	IV

**Table 8:** Five levels and the corresponding elements for GTA.

Level	Elements
L1 (Level 1)	E7
L2 (Level 2)	E3
L3 (Level 3)	E1, E4, E6
L4 (Level 4)	E5
L5 (Level 5)	E2

## Data Availability

Not applicable.
